# Beneficial Effects of Fibroblast Growth Factor-1 on Retinal Pigment Epithelial Cells Exposed to High Glucose-Induced Damage: Alleviation of Oxidative Stress, Endoplasmic Reticulum Stress, and Enhancement of Autophagy

**DOI:** 10.3390/ijms25063192

**Published:** 2024-03-11

**Authors:** Hsin-Wei Huang, Chung-May Yang, Chang-Hao Yang

**Affiliations:** 1 Department of Ophthalmology, Wan Fang Hospital, Taipei Medical University, No. 111, Section 3, Xinglong Road, Taipei 11696, Taiwan; 100320@w.tmu.edu.tw; 2 Graduate Institute of Clinical Medicine, College of Medicine, National Taiwan University, No. 1, Jen Ai Road Section 1, Taipei 100, Taiwan; 3 Department of Ophthalmology, National Taiwan University Hospital, No. 7, Zhongshan South Road, Taipei 100, Taiwan; chungmay@ntu.edu.tw; 4 College of Medicine, National Taiwan University, No. 1, Jen Ai Road Section 1, Taipei 100, Taiwan

**Keywords:** fibroblast growth factor-1, diabetic retinopathy, oxidative stress, endoplasmic reticulum stress, autophagy, adenosine monophosphate-activated protein kinase, mammalian target of rapamycin

## Abstract

Diabetic retinopathy (DR) severely affects vision in individuals with diabetes. High glucose (HG) induces oxidative stress in retinal cells, a key contributor to DR development. Previous studies suggest that fibroblast growth factor-1 (FGF-1) can mitigate hyperglycemia and protect tissues from HG-induced damage. However, the specific effects and mechanisms of FGF-1 on DR remain unclear. In our study, FGF-1-pretreated adult retinal pigment epithelial (ARPE)-19 cells were employed to investigate. Results indicate that FGF-1 significantly attenuated HG-induced oxidative stress, including reactive oxygen species, DNA damage, protein carbonyl content, and lipid peroxidation. FGF-1 also modulated the expression of oxidative and antioxidative enzymes. Mechanistic investigations showed that HG induced high endoplasmic reticulum (ER) stress and upregulated specific proteins associated with apoptosis. FGF-1 effectively alleviated ER stress, reduced apoptosis, and restored autophagy through the adenosine monophosphate-activated protein kinase/mammalian target of the rapamycin signaling pathway. We observed that the changes induced by HG were dose-dependently reversed by FGF-1. Higher concentrations of FGF-1 (5 and 10 ng/mL) exhibited increased effectiveness in mitigating HG-induced damage, reaching statistical significance (*p* < 0.05). In conclusion, our study underscores the promising potential of FGF-1 as a safeguard against DR. FGF-1 emerges as a formidable intervention, attenuating oxidative stress, ER stress, and apoptosis, while concurrently promoting autophagy. This multifaceted impact positions FGF-1 as a compelling candidate for alleviating retinal cell damage in the complex pathogenesis of DR.

## 1. Introduction

Diabetes mellitus (DM) is a complex metabolic disorder marked by hyperglycemia and insulin resistance [1]. Diabetic retinopathy (DR) not only causes severe vision impairment in patients with DM but also imposes a considerable economic burden on patients and the health care system [2]. Although the pathophysiology of DR is partly understood to a certain degree, the specific molecular mechanisms remain incompletely elucidated. The current treatment strategies for DR (e.g., retinal laser photocoagulation, intravitreal injection of antivascular endothelial growth factor agents or steroids, and vitrectomy) have not been upgraded for a long period of time [3]. Therefore, it is crucial to develop novel strategies for the prevention and treatment of DR.

DM increases oxidative stress, which results in cell damage mediated by reactive oxygen species (ROS) and impaired antioxidant capacity [4]. Our previous research demonstrated markedly increased ROS production in the vitreous humor of patients with proliferative DR. Furthermore, the levels of ROS were found to be correlated with the severity of proliferative DR [5].

In addition to oxidative stress, endoplasmic reticulum (ER) stress plays a key role in various physical diseases. Physiological stresses, such as DM, disrupt the balance between protein folding demand and ER protein folding capacity, leading to an imbalance that induces ER stress. This ER stress is a potential mediator of retinal inflammation and is associated with the pathophysiology of DR [6,7,8,9]. Apoptosis is integral to DR progression [10]. High glucose (HG) levels contribute to increased production of ROS and promote the apoptosis of retinal pigment epithelial (RPE) cells [11].

Autophagy, an intracellular lysosome-related degradation process, helps maintain the cellular balance between the synthesis and degradation of cellular components; it serves as a protective mechanism against physiological senescence and apoptosis [12]. The regulation of autophagy is affected by various cellular stress conditions, such as oxidative stress, ER stress, nutrient and growth factor deficiency, and infection [13,14]. In the context of the eyes, the autophagic process is essential for maintaining normal physiological function. It is involved in the renewal of the outer segment of photoreceptors and the protection of RPE cells against oxidative stress and melanin degradation [15,16]. Moreover, the dysregulation of autophagy is also implicated in the pathophysiology of DR [17].

Fibroblast growth factor-1 (FGF-1) is an autocrine and paracrine regulatory molecule that has been extensively studied due to its ability to induce mitotic activity in various cells, including heart [18], liver [19], kidney [20], vasculature [21], and skin [22] cells. Researchers have focused on the therapeutic benefits of FGF-1 in cardiovascular disorders [23], nerve injury, and wound healing [24]. In addition to its well-known traditional role in promoting mitosis, FGF-1 has been found to have various intriguing functions. FGF-1 has also shown other capabilities, such as enhancing glucose uptake in an insulin-dependent manner [25] and reducing glucose levels in an insulin-independent manner [26], to achieve metabolic euglycemia.

In addition to its ability to lower blood glucose levels without inducing hypoglycemia, FGF-1 exhibits promising potential in the treatment of complications associated with diabetes. For instance, FGF-1 can reduce inflammation caused by diabetes, lower oxidative stress, resist cell apoptosis, and enhance cellular autophagy. FGF-1 has been shown to reduce oxidative stress and endoplasmic reticulum (ER) stress in diabetic nephropathy [27,28,29]. Furthermore, FGF-1 ameliorates diabetic cardiomyopathy through the alleviation of oxidative stress [30] and anti-apoptotic effects [31]. FGF-1 has also been found to alleviate diabetes-induced mouse germ cell death by decreasing ER stress and apoptotic cell death [32]. Additionally, FGF-1 inhibits diabetes-induced liver injury by decreasing oxidative stress [33] and restoring autophagy [34].

The retinal pigment endothelium (RPE) is a single layer of cells located between the retina and choroid. Its primary role is to support the metabolic nutrition of retinal photoreceptor cells [35]. The dysfunction of these RPE cells is closely linked to the pathophysiology and progression of DR [36]. Adult RPE (ARPE)-19 cells possess structural and functional properties that closely resemble RPE cells in vivo. ARPE-19 cells cultured with HG are often used to simulate DM, and they serve as an ideal in vitro model for DR studies [11,37].

In the present study, our hypothesis was that treatment with FGF-1 would alleviate oxidative stress and ER stress induced by HG in ARPE-19 cells. Additionally, we aimed to investigate whether FGF-1 could reduce apoptosis and restore autophagy in these cells. We further explored the mechanisms underlying the effects of FGF-1 on these cellular processes.

## 2. Results

### 2.1. FGF-1 Mitigates High Glucose-Induced Oxidative Stress by Suppressing Oxidative Enzyme Expression and Enhancing Antioxidative Enzyme Expression

**Figure 1:**
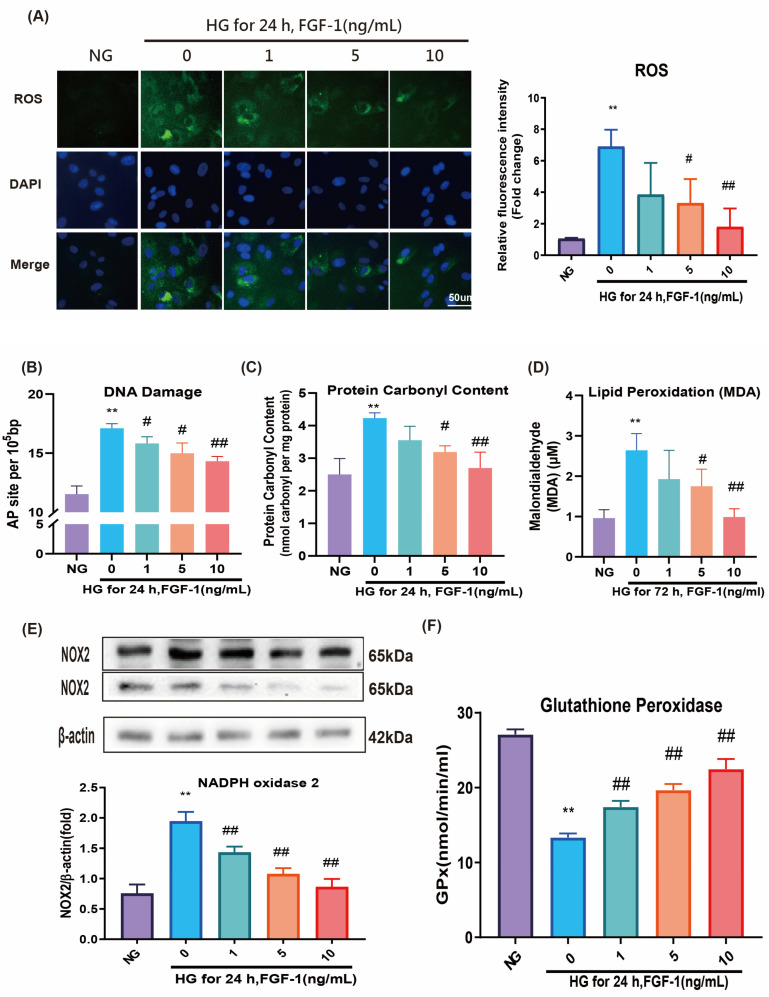
FGF-1 blocked High Glucose (HG)-induced oxidative stress. ARPE-19 cells were pretreated with various concentrations of FGF-1 (1, 5, and 10 ng/mL) for 2 h and then incubated in HG (25 mM) medium for 24 or 72 h. (**A**) DCFDA fluorescence staining was performed to measure ROS levels. The relative density levels of DCFDA fluorescence were quantified using ImageJ (version 1.54d; NIH, Bethesda, MD, USA) and presented in bar graphs. (*n* = 5 for each group). Bar = 50 µm. (**B**) DNA damage (AP sites) was measured using a kit. (**C**) Protein oxidation was measured using a protein carbonyl content assay kit. (**D**) Lipid peroxidation (indicated by malondialdehyde level) was measured using a TBARS assay kit. (**E**) Western blotting was performed to measure NOX2 expression. (**F**) An enzyme-linked immunosorbent assay was performed to measure GPx expression. (*n* = 5 for each group). Data are presented as mean ± standard deviation values; ** *p* < 0.01 versus cells incubated in normal glucose (NG) medium (control); # *p* < 0.05, ## *p* < 0.01 versus ARPE-19 cells incubated in HG medium. For the comparison of numerical data among the groups, we employed either one-way ANOVA or the Kruskal–Wallis test, followed by post hoc testing.

Oxidative stress is a hallmark of complications associated with elevated levels of high glucose (HG). In this study, we employed dichlorodihydrofluorescein diacetate (DCFDA) fluorescence staining to investigate the impact of HG on ROS levels in ARPE-19 cells. Our results demonstrated a significant increase in ROS levels in ARPE-19 cells cultured in HG medium. Notably, treatment with FGF-1, particularly at high concentrations (5 and 10 ng/mL), substantially attenuated these elevated ROS levels (Figure 1A).

To further assess oxidative stress, we evaluated three key biomarkers in ARPE-19 cells: the AP (apurinic/apyrimidinic) sites indicative of DNA peroxidation products, protein carbonyl content, and the extent of lipid peroxidation (LPO), reflected by malondialdehyde levels. All three markers of oxidative stress were significantly elevated in ARPE-19 cells cultured in HG medium (Figure 1B–D), consistent with the observed increase in ROS expression (evidenced by an immunofluorescence increase; Figure 1A). However, the adverse effects induced by HG were mitigated in a concentration-dependent manner by the addition of FGF-1 (Figure 1B–D).

Additionally, Western blotting (WB) analysis revealed a notable increase in the expression level of the oxidative enzyme nicotinamide adenine dinucleotide phosphate oxidase 2 (NOX2) under HG conditions (Figure 1E). The application of FGF-1 showed the ability to reduce the elevated NOX2 level, with higher concentrations of FGF-1 exhibiting greater effectiveness in mitigating the HG-induced NOX2 increase.

Furthermore, results from the enzyme-linked immunosorbent assay (ELISA) revealed that HG had an adverse impact on the expression level of the antioxidative enzyme glutathione peroxidase (GPx) (Figure 1F), which could be substantially restored by the presence of FGF-1. Notably, higher concentrations of FGF-1 exhibited a more pronounced ability to elevate GPx levels.

In summary, these results provided compelling evidence that FGF-1 effectively alleviates HG-induced oxidative stress, suppresses the expression of NOX2, and increases the expression of GPx in ARPE-19 cells. These findings may indicate the potential of FGF-1 as a therapeutic agent against oxidative stress.

### 2.2. FGF-1 Alleviates ER Stress Induced by High Glucose

**Figure 2:**
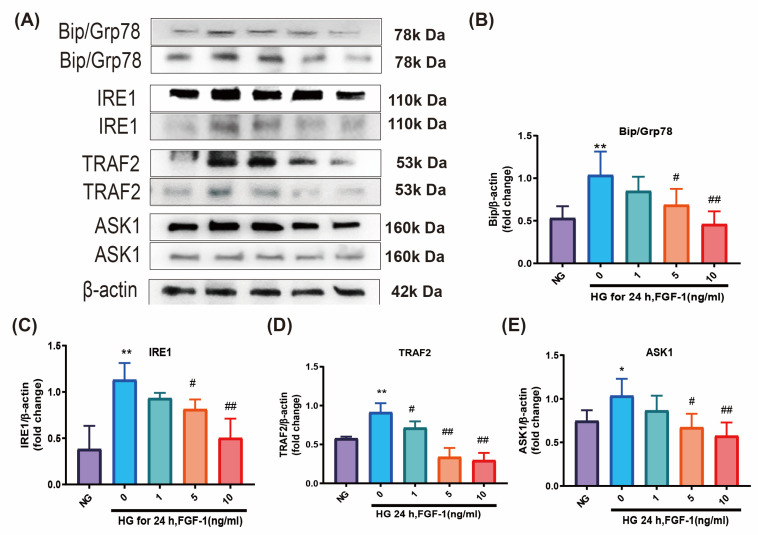

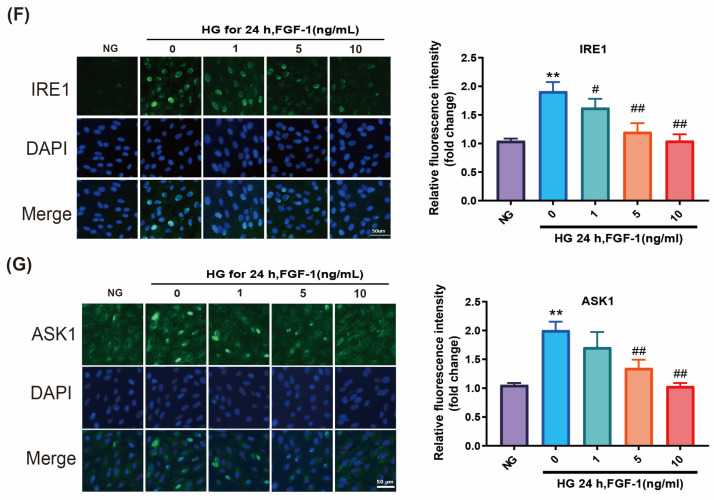
FGF-1 alleviated ER stress in ARPE-19 cells incubated in HG medium. Expression levels of ER stress mediators were evaluated through Western blotting: (**A**) BiP/Grp78, IRE1, TRAF2, and ASK1. Loading control images are re-used for illustrative purposes. Statistical analysis of protein expression of (**B**) BiP/Grp78, (**C**) IRE1, (**D**) TRAF2, and (**E**) ASK1 in Western blotting. Immunofluorescence imaging results for (**F**) IRE1 and (**G**) ASK1 were performed. Immunofluorescence image relative densities were assessed through the utilization of ImageJ software (version 1.54d) and subsequently illustrated using bar graphs. (*n* = 5 for each group). * *p* < 0.05, ** *p* < 0.01 versus cells incubated in NG medium (control); # *p* < 0.05, ## *p* < 0.01 versus ARPE-19 cells incubated in HG medium. For the comparison of numerical data among the groups, we utilized either one-way ANOVA or the Kruskal–Wallis test, followed by post hoc testing.

ER stress plays a significant role in the pathophysiology of DR [6,7,8,9]. ER stress blockers are anticipated to emerge as a novel therapy for DR. In this study, we conducted WB to investigate the expression of ER stress markers, including immunoglobulin-binding protein/glucose-regulated protein 78 (BiP/Grp78), inositol-requiring protein-1 (IRE1), tumor necrosis factor-associated factor 2 (TRAF2), and anti-apoptosis signal-regulating kinase 1 (ASK1) in ARPE-19 cells. The levels of these four ER stress markers were notably elevated in cells cultured in HG medium (Figure 2A–E). FGF-1 reduced the expression levels of these markers, and higher concentrations of FGF-1 exhibited greater effectiveness in mitigating HG-induced ER stress.

To further confirm our findings, we conducted immunofluorescence imaging to assess the levels of IRE1 and ASK1 in ARPE-19 cells (Figure 2F,G). Immunofluorescence signals for IRE1 and ASK1 were notably increased in ARPE-19 cells cultured in HG medium, but these HG-induced changes were dose-dependently reversed by FGF-1. Higher concentrations of FGF-1 (5 and 10 ng/mL) demonstrated greater efficacy. In summary, our findings provide evidence that FGF-1 effectively alleviates HG-induced ER stress in ARPE-19 cells, highlighting its potential as a therapeutic intervention for DR.

### 2.3. FGF-1 Reduced HG-Induced Apoptosis and Ameliorated Mitochondrial Dysfunction

**Figure 3:**
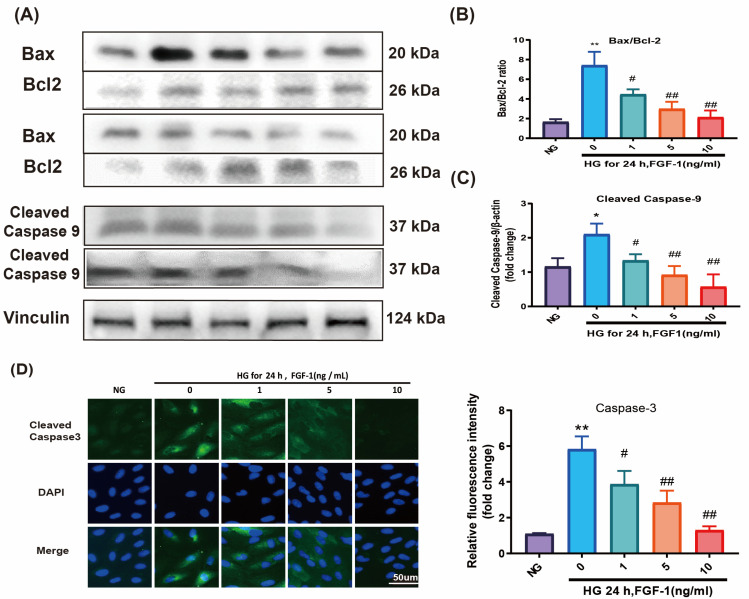

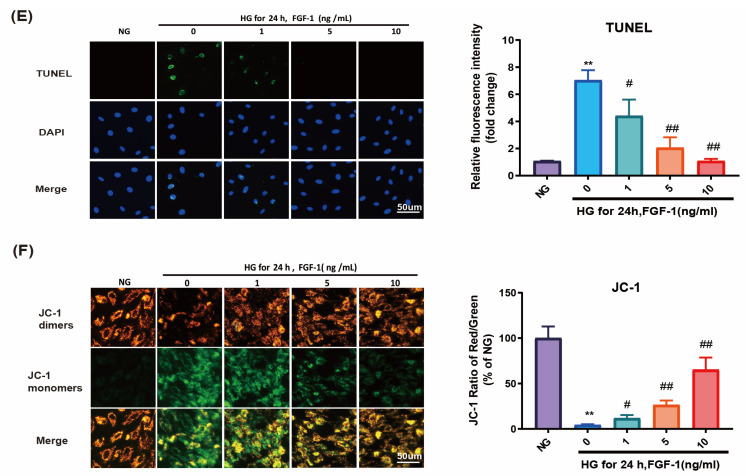
FGF-1 alleviates apoptosis induced by high glucose and improves mitochondrial dysfunction. Western blotting was employed to assess the expression levels of apoptotic mediators, including (**A**) Bax/Bcl-2 and caspase-9. Loading control images were reused for illustrative purposes. Statistical analysis of protein expression of (**B**) Bax/Bcl-2 and (**C**) caspase-9 in Western blotting. Results from (**D**) caspase-3 immunofluorescence imaging, (**E**) TUNEL assay, and (**F**) JC-1 staining are presented. Immunofluorescence image relative densities were assessed through the utilization of ImageJ software and subsequently illustrated using bar graphs. (*n* = 5 for each group). * *p* < 0.05, ** *p* < 0.01, compared to cells incubated in normal glucose (NG) medium (control); # *p* < 0.05, ## *p* < 0.01, compared to ARPE-19 cells incubated in high glucose (HG) medium. For the comparison of numerical data among the groups, we utilized either one-way ANOVA or the Kruskal–Wallis test, followed by post hoc testing.

To elucidate the protective mechanisms of FGF-1 against diabetic retinopathy, we investigated apoptotic pathways. Western blot analysis revealed a significantly elevated Bax/Bcl-2 ratio in ARPE-19 cells cultured in HG medium compared to those cultured in NG medium (Figure 3A,B).

Caspase-9 and caspase-3 play essential roles in facilitating the intrinsic apoptotic pathways [38]. ARPE-19 cells cultured in a HG medium exhibited elevated levels of cleaved caspase-9 proteins in comparison to ARPE-19 cells cultured in a NG medium (Figure 3A,C). These HG-induced changes were effectively reversed by the presence of FGF-1. To validate the expression of caspase-3 in ARPE-19 cells, immunofluorescence imaging was performed (Figure 3D). The results confirmed heightened levels of caspase-3 expression in ARPE-19 cells cultured in HG medium, and this increase was concentration-dependently attenuated by FGF-1.

We also assessed apoptotic damage using a terminal deoxynucleotidyl transferase dUTP nick end labeling (TUNEL) assay. A significantly higher number of ARPE-19 cells cultured in HG medium showed signs of apoptosis compared to those cultured in NG medium (Figure 3E). Notably, FGF-1, especially at high concentrations (5 and 10 mg/mL), decreased the number of TUNEL-positive cells.

Mitochondrial membrane potential dysfunction was evaluated using the JC-1 dye. In an HG environment, the dye emitted green fluorescence (JC-1 monomers), indicating pronounced mitochondrial dysfunction. In contrast, after FGF-1 treatment, the dye formed aggregates in normal cells (JC-1 dimers) and exhibited red fluorescence, signifying the health of the examined cells (Figure 3F). The results imply that FGF-1 reduces apoptosis induced by HG and ameliorates mitochondrial dysfunction in ARPE-19 cells.

### 2.4. FGF-1-Restored Autophagy in HG-Affected ARPE-19 Cells

**Figure 4:**
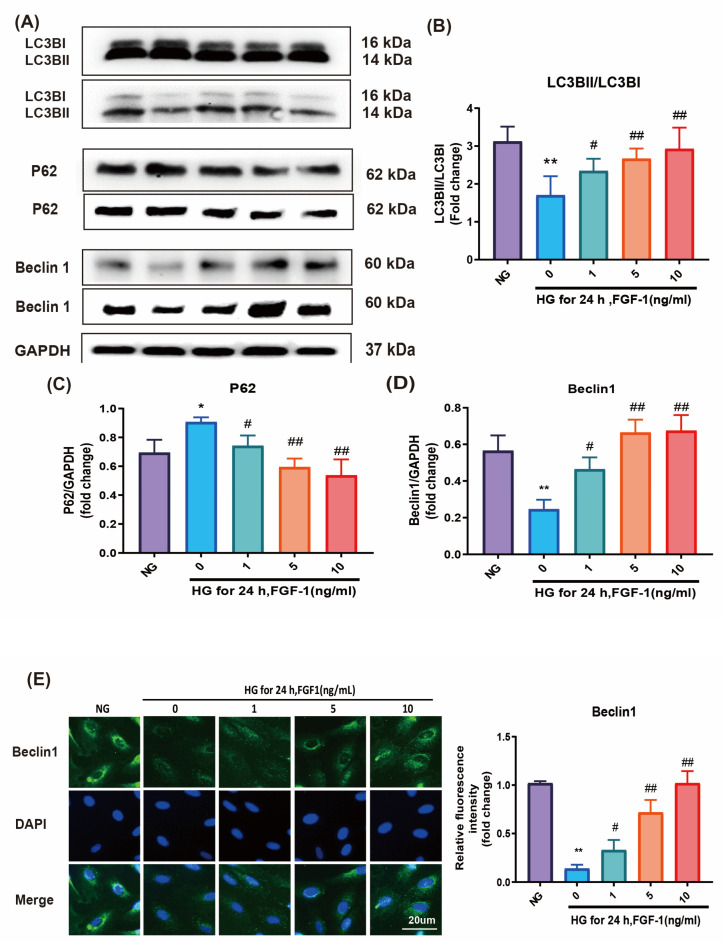
FGF-1 restored autophagy in ARPE-19 cells cultured in HG medium. The (**A**) LC3BII/LC3BI ratio, P62, and Beclin-1 expression levels were determined through Western blotting. GAPDH served as a loading control, and the image was reused for illustrative purposes. Statistical analysis of protein expression of (**B**) LC3BII/LC3BI ratio, (**C**) P62, and (**D**) Beclin 1 in Western blotting. (**E**) Immunofluorescence imaging results for Beclin 1. Immunofluorescence image relative densities were assessed through the utilization of ImageJ software and subsequently illustrated using bar graphs. (*n* = 5 for each group). * *p* < 0.05, ** *p* < 0.01 versus cells incubated in NG medium (control); # *p* < 0.05, ## *p* < 0.01 versus ARPE-19 cells incubated in HG medium. For the comparison of numerical data among the groups, we utilized either one-way ANOVA or the Kruskal–Wallis test, followed by post hoc testing.

To investigate the potential of FGF-1 in restoring autophagy, we assessed various autophagy markers, including the microtubule-associated protein 1 light chain 3B II/I (LC3BII/LC3BI) ratio, P62, and Beclin 1. The LC3BII/LC3BI ratio and Beclin 1 levels were diminished in ARPE-19 cells cultured in HG medium, and these effects were reversed by FGF-1 treatment (Figure 4A,B,D). In contrast, the level of P62 was elevated in ARPE-19 cells cultured in HG medium compared to those in NG medium. As anticipated, FGF-1 treatment reduced the level of P62 in ARPE-19 cells (Figure 4A,C). We further assessed the expression of Beclin 1 through an immunofluorescence assay (Figure 4E). The results were consistent with the Western blot analysis, demonstrating that FGF-1 mitigated the HG-induced reductions in autophagy markers. Collectively, these findings suggest that FGF-1 effectively restores autophagy in ARPE-19 cells affected by high glucose.

### 2.5. FGF-1-Enhanced AMPK/mTOR Signaling in HG-Affected ARPE-19 Cells

**Figure 5:**
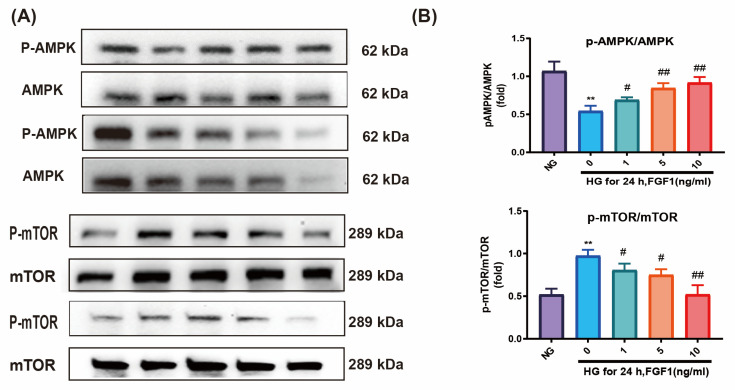

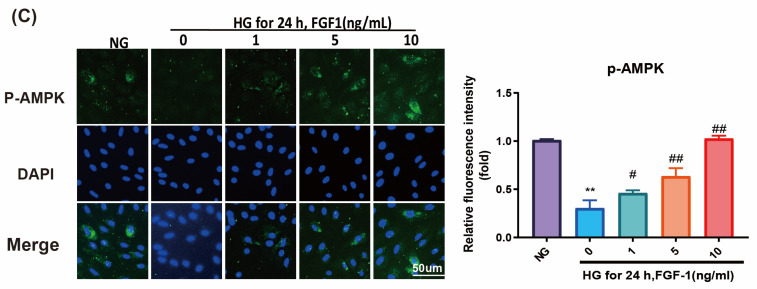
FGF-1 enhanced AMPK/mTOR signaling in ARPE-19 cells cultured in HG medium. (**A**) Western blot analysis of phospho-AMPK/AMPK and phospho-mTOR/mTOR expression. (**B**) Statistical analysis of p-AMPK/AMPK and p-mTOR/mTOR expressions in Western blotting. (**C**) Immunofluorescence imaging of phospho-AMPK. Immunofluorescence image relative densities were assessed through the utilization of ImageJ software and subsequently illustrated using bar graphs. (*n* = 5 for each group). ** *p* < 0.01 versus cells incubated in normal glucose (NG) medium (control); # *p* < 0.05, ## *p* < 0.01 versus ARPE-19 cells incubated in HG medium. For the comparison of numerical data among the groups, we utilized either one-way ANOVA or the Kruskal–Wallis test, followed by post hoc testing.

Western blot analysis revealed a decrease in phospho-AMPK levels and an increase in phospho-mTOR levels in ARPE-19 cells cultured in HG medium compared to those in NG medium. Treatment with FGF-1 resulted in the upregulation of phospho-AMPK expression and a concurrent downregulation of phospho-mTOR levels in ARPE-19 cells affected by HG (Figure 5A,B). To validate the consistency of these trends observed in Western blot results, we further confirmed the expression of phospho-AMPK through an immunofluorescence assay (Figure 5C). In summary, these findings suggest that the AMPK/mTOR pathway mediates the effects of FGF-1 on ARPE-19 cells exposed to high glucose, influencing both the inhibition of apoptosis and the induction of autophagy.

## 3. Discussion

DR stands as a prominent cause of vision impairment in patients with diabetes mellitus worldwide. Despite its prevalence, there is a scarcity of developed treatment strategies for this significant ailment. Identifying new therapeutic targets and creating effective drugs are imperative for the successful management of DR. In this study, we observed substantial apoptosis induced by oxidative stress and ER stress in an in vitro DR model induced by HG. However, FGF-1 demonstrated the capability to alleviate HG-induced oxidative stress and ER stress, mitigate apoptosis, and restore autophagy by enhancing AMPK/mTOR signaling in ARPE-19 cells. Consequently, FGF-1 emerges as a promising therapeutic target for DR.

Initially recognized as a mitogen and isolated from bovine pituitary glands [39], FGF-1 has since been extensively studied and employed in treating cardiovascular disease, nerve damage, and wound healing [23]. Numerous studies have highlighted the benefits of FGF-1 for individuals with DM, as it helps with blood glucose homeostasis and insulin sensitization [40,41,42]. Additionally, FGF-1 has shown efficacy in preventing diabetic nephropathy by mitigating renal inflammation with a reduction in nuclear factor κB and c-Jun N-terminal kinase signaling pathways [27] and via the PI3K/AKT pathway in different studies [28]. Furthermore, FGF-1 has demonstrated its ability to improve DM-induced hepatic steatosis in leptin-deficient ob/ob mice through its anti-inflammatory function [33].

Notably, patients with nonproliferative DR exhibit significantly lower levels of FGF-1 in their aqueous humor compared to healthy individuals [43]. Thus, our hypothesis posits that elevating FGF-1 levels in the eyes could shield retinas from diabetic HG damage. However, the molecular mechanisms underlying the effects of FGF-1 remain unclear, particularly its associations with ER stress, apoptosis, and autophagy, warranting further investigation.

Oxidative stress is a key contributor to DM-related complications, including diabetic cardiomyopathy, kidney disease, and liver damage [44]. FGF-1 mitigates oxidative stress, preventing diabetic cardiomyopathy [30,45], kidney cell stress in diabetic nephropathy [29,46], and cellular stress in DM-induced liver injury [34,47]. In our study, we confirmed that FGF-1 induces antioxidative stress in HG-affected cells by inhibiting ROS overproduction and alleviating oxidative stress.

The NOX enzyme’s activity increases during ROS generation in various nonphagocytic cells, and its association with DM has been established [48]. In our study, FGF-1 significantly downregulated the expression of NOX2. Additionally, FGF-1 exhibited pronounced suppression of DM-induced NOX2 overexpression in db/db mouse kidney [29] and spleen [49] cells. GPx serves as an enzymatic scavenging antioxidant responsive to oxidative stress, and a decline in GPx antioxidant defense is linked to DR progression [50]. Our results demonstrated that FGF-1 substantially augmented the antioxidant defense system, specifically the GPx level, in ARPE-19 cells cultured in HG medium. In a relevant study, FGF-1 not only inhibited acetaminophen-induced oxidative stress but also increased GPx levels in mouse liver cells [51]. Thus, our findings confirm that FGF-1 reduces the expression of the oxidative enzyme NOX2 while concurrently increasing the antioxidative enzyme GPx in HG-affected ARPE-19 cells.

The ER is a crucial organelle essential for maintaining normal cellular physiological activities. The accumulation of unfolded and misfolded proteins in the ER triggers ER stress, potentially resulting in the loss of ER function and apoptosis. The Unfolded Protein Response (UPR) is the cell’s mechanism to mitigate these undesirable effects [52]. The redox capacity of the ER determines the fate of these proteins, and the levels of redox mediators regulate the generation of reactive oxygen species (ROS). Sustained UPR and ER stress induce a ROS cascade, leading to oxidative stress and various human disorders, including DM, neurodegenerative diseases, inflammation, kidney, and liver diseases [53,54]. Studies have suggested the involvement of ER stress in retinal inflammation and the pathophysiology of DR [6,7,8].

ER stress is mediated through three signaling transduction proteins—IRE1, activating transcription factor 6 (ATF6), and protein kinase RNA-like ER kinase (PERK)—which are regulated by BiP/Grp78 [55]. IRE1 can form a complex with TRAF2, recruiting ASK1. The IRE1–TRAF2–ASK1 complex is strongly associated with neural cell death [56] and cellular caspase- and mitochondria-dependent apoptosis [57,58].

In our present study, we observed significantly elevated levels of ER stress-related proteins, including BiP/Grp78, IRE1, TRAF2, and ASK1, in ARPE-19 cells affected by high glucose compared to unaffected cells. These changes were associated with apoptosis and were effectively reversed by FGF-1. FGF-1 has demonstrated its ability to alleviate ER stress in various tissues by acting on different molecules. For instance, in the testicular cells of diabetic mice, FGF-1 markedly reduced the expression levels of BiP/Grp78 and C/EBP-homologous protein (CHOP) [32]. Similarly, it reduced the expression levels of BiP/GRP78, IRE1, ATF6, PERK, and CHOP in the kidney cells of diabetic mice [29]. Additionally, FGF-1 inhibited the expression of BiP/GRP78, IRE1, ATF6, PERK, CHOP, and Eif2a in the liver cells of diabetic mice [34]. The findings from our study and previous research collectively indicate that FGF-1 effectively protects against high glucose toxicity by alleviating ER stress.

Excessive apoptosis is a key molecular mechanism contributing to complications in DM [59]. HG activates various apoptosis-related proteins, including mitogen-activated protein kinases (e.g., P38, JNK, and extracellular signal-regulated kinase), members of the Bcl-2 family, and caspases [59,60]. Within the Bcl-2 family, Bax and Bcl-2 play pivotal roles. Bax, a proapoptotic protein, promotes apoptosis by permeabilizing the outer mitochondrial membrane in response to various oxidative stress conditions. In contrast, Bcl-2, an anti-apoptotic protein, suppresses Bax activity to prevent apoptosis [61]. Following the release of cytochrome c from mitochondria, the caspase cascades of caspase-9 and caspase-3 activate the oxidative stress-mediated intrinsic apoptotic pathway [59,62,63].

In our study, HG increased the Bax/Bcl-2 ratio and upregulated the expression of apoptosis-related proteins, including cleaved caspase-9 and caspase-3. These effects were effectively reversed by FGF-1. Additionally, TUNEL and JC-1 assays demonstrated that FGF-1 reduced HG-induced apoptosis and mitigated mitochondrial dysfunction in ARPE-19 cells in a concentration-dependent manner. FGF-1 has shown efficacy in ameliorating DM-induced apoptosis in various tissues, including myocardial apoptosis [31], hepatic apoptosis [34,51], renal apoptosis [27,49], and testicular apoptosis [32]. The combined findings from our present study and previous research highlight FGF-1’s protective role against HG-induced apoptosis in ARPE-19 cells.

Autophagy, a crucial homeostatic process for intracellular degradation, involves the conversion of LC3-I into LC3-II, with the LC3BII/LC3BI ratio commonly used as a marker for autophagosome formation [64]. Beclin 1 is vital for the localization of autophagic proteins to the preautophagosomal structure, playing an essential role in autophagy onset and serving as a marker for monitoring autophagy [65]. Additionally, the ubiquitin-binding protein P62, also known as sequestosome 1 (SQSTM1), is linked to protein degradation and acts as a negative marker of autophagy [66]. During autophagy, P62 binds to ubiquitinated proteins in the cytoplasm, then localizes with LC3-II on the autophagosome and is eventually degraded in autolysosomes. Because the P62 protein is continuously degraded as autophagy progresses, its expression is inversely correlated with autophagy [67].

In our study, FGF-1 enhanced the protective effect of autophagy on ARPE-19 cells by regulating autophagy-related proteins. FGF-1 increased the LC3BII/LC3BI ratio. Furthermore, the elevated expression level of the autophagy-related protein Beclin-1 indicated that FGF-1 promoted autophagy in ARPE-19 cells. Conversely, FGF-1 decreased the expression level of P62 in HG-affected ARPE-19 cells. Autophagy is recognized as a double-edged sword in the pathophysiology of diabetic retinopathy [68]. Under mild or short-term stress, autophagy can promote cell survival by eliminating misfolded proteins, unfolded proteins, and damaged organelles and by inhibiting caspase activation; however, under severe or long-term stress, impaired autophagy can lead to apoptosis [69]. Consistent with our findings, other studies have reported considerable impairment of mitophagy in ARPE-19 cells under high glucose conditions [11] and reduced autophagy in rat retinal Müller cells [70]. Furthermore, a study indicated that FGF-1 restored defective hepatic autophagy in db/db mice [34]. Thus, our study confirms that FGF-1 restores autophagy in HG-affected ARPE-19 cells.

The molecular mechanisms underlying the effects of FGF-1 on ARPE-19 cells are not fully understood. Autophagy appears to play a protective role by modulating the PTEN-induced putative kinase 1/Parkin signal pathway [11,71]. Previous studies have highlighted the dual role of mTOR, which not only regulates apoptosis [72] but also inhibits autophagy [73]. AMPK, a key sensor of nutrition, energy, and stress, modulates cellular processes by upregulating catabolism and downregulating anabolism [74]. Additionally, AMPK inhibits mTOR activity, thereby suppressing apoptosis and promoting autophagy [75].

Our findings demonstrate that FGF-1 upregulates AMPK phosphorylation and downregulates mTOR phosphorylation in HG-affected ARPE-19 cells, resulting in reduced apoptosis and enhanced autophagy. Consistent with our results, other studies have reported that FGF-1 significantly increases AMPK phosphorylation, preventing nonalcoholic fatty liver disease [47] and diabetic cardiomyopathy [45] in mice.

FGF-1 may also modulate autophagy by influencing interactions between the autophagic protein Beclin 1 and members of the anti-apoptotic Bcl-2 family [76]. In our study, FGF-1 enhances autophagy by increasing Beclin 1 expression and reduces apoptosis by elevating Bcl-2 expression. The Bcl-2/Beclin 1 complex, as highlighted in a review [77], plays a pivotal role in the interplay between autophagy and apoptosis, where autophagy negatively regulates apoptosis through the interaction between Bcl-2 and Beclin 1 [69].

**Figure 6:**
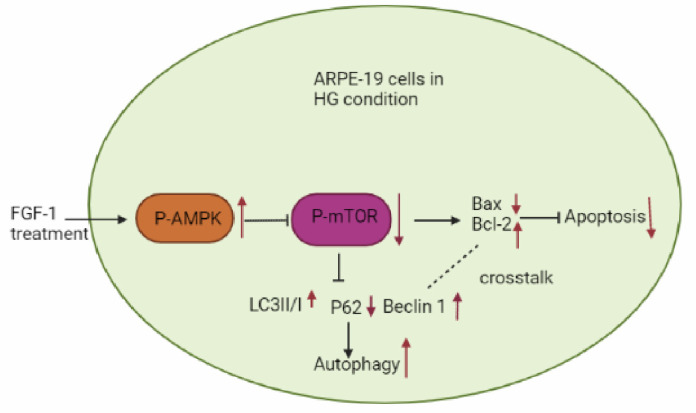
Protective effects of FGF-1 on ARPE-19 cells through the AMPK/mTOR signaling pathway. Black arrows represent facilitation, black broken arrows represent inhibition, red upward arrows represent increases, red downward arrows represent decreases.

Studies have reported that increased ER stress-oxidative stress, and impaired antioxidant defense may contribute to diabetic heart disease [78]. Autophagy, acting as a protective mechanism, serves as a recycling pathway to eliminate impaired proteins or pathogens, thereby maintaining cell health. ER stress and oxidative stress significantly regulate autophagy. Previous reports have suggested that compounds like salidroside can promote autophagy and reduce oxidative stress by activating the AMPK pathway and downregulating the mTOR pathway in HUVECs [79]. Bilberry anthocyanins have been shown to activate autophagy and reduce oxidative stress by inducing phosphorylation of AMPK and FOXO3a and reducing p-mTOR in rats [80]. Therefore, we propose that FGF-1 ameliorates ER stress and oxidative stress, exerting autophagic and anti-apoptotic effects by activating the AMPK/mTOR signaling pathway in HG-affected ARPE-19 cells (Figure 6).

## 4. Materials and Methods

### 4.1. Cell Culture and Experimental Design

Human ARPE-19 cells were obtained from the American Type Culture Collection (Manassas, VA, USA). These cells were cultured in Dulbecco’s Modified Eagle Medium/Nutrient Mixture F-12 (11320033; Invitrogen, Carlsbad, CA, USA) supplemented with 10% fetal bovine serum (26140079; Invitrogen, Carlsbad, CA, USA) and a solution of 100 U/mL penicillin (B13233; Invitrogen, Carlsbad, CA, USA) and 100 μg/mL streptomycin (11860038; Invitrogen, Carlsbad, CA, USA). The cell cultures were maintained in a cell incubator at 37 °C with a 5% CO_2_ atmosphere. Only cells from passages three to five were utilized in subsequent experiments.

For the experiments, ARPE-19 cells were cultivated in either normal glucose (NG; 5 mM) or high-glucose (HG; 25 mM) medium [81,82,83]. Additionally, the cells were pre-treated with 0, 1, 5, or 10 ng/mL of FGF-1 (Catalog Number 232-FA; R&D Systems, Minneapolis, MN, USA) for 2 h before exposure to HG medium. FGF-1 was intentionally retained in the medium during the treatment of cells with HG. After 24 or 72 h of HG treatment, the cells were harvested for analysis.

To investigate whether the impact of high glucose was related to osmolarity (Supplementary Figures S1–S3), ARPE-19 cells were exposed to an osmotic control medium for 24 h. This medium had a concentration selected to mimic the osmolarity of 25 mM glucose, achieved by culturing the cells in 5 mM glucose supplemented with 20 mM mannitol (M4125 Sigma-Aldrich, St. Louis, MO, USA). Mannitol was used to exclude the influence of osmotic pressure [84].

### 4.2. Measurement of Intracellular ROS Levels

We used 2′,7′-dichlorodihydrofluorescein diacetate (2′,7′-DCFDA; Sigma-Aldrich, St. Louis, MO, USA) to assess intracellular ROS levels in ARPE-19 cells exposed to various concentrations of FGF-1 [85]. The cells were incubated with a 40 μM fluorescent probe known as 2′,7′-dichlorofluorescein diacetate (H_2_DCFDA) for 30 min at 37 °C. H_2_DCFDA, a non-ionic probe, easily permeates ARPE-19 cells and undergoes deacetylation by intracellular esterases. This enzymatic reaction converts H_2_DCFDA into a non-fluorescent compound, 2′,7′-dichlorodihydrofluorescein (DCFH), which is subsequently oxidized to the fluorescent 2′,7′-dichlorofluorescein (DCF) in the presence of intracellular ROS. The vibrant green fluorescence emitted by DCF was quantified using a microplate reader (Bio-Rad Laboratories, Hercules, CA, USA) with excitation at 485 nm and emission at 525 nm wavelengths.

To calculate the ROS immunostaining expression, we used the following formula: Immunostaining index = Σ [(X − threshold) × area (in pixels)]/total cell count, where X represents staining density, indicated as a value ranging from 0 to 256 in grayscale. In essence, digitized color images were acquired as PICT files, and these files were opened in grayscale mode using ImageJ software (version 1.54d; NIH, Bethesda, MD, USA). We analyzed five images per experiment, with normal glucose ARPE-19 cells serving as the normal reference.

### 4.3. Assessment of DNA Oxidative Damage

Genomic DNA was extracted from ARPE-19 cells using the Genomic DNA Isolation Kit (ab65358; Abcam, Cambridge, MA, USA). The quantification of AP sites in the isolated DNA was performed with the DNA Damage Assay Kit (ab211154; Abcam, Cambridge, MA, USA) following the manufacturer’s instructions. This colorimetric assay measures AP sites [86], a prominent indicator of DNA damage, by reacting with aldehyde groups on open-ring AP sites. Absorbance was determined at OD 450 nm using a microplate reader. Standard absorbance values were plotted in Microsoft Excel to determine AP sites per 10^5^ base pairs. The most accurate trendline equation was calculated from the standard absorbance curve data. Sample OD readings were then applied to the standard curve equation to determine the number of AP sites per 10^5^ base pairs for each sample.

### 4.4. Determination of Protein Oxidation

Protein carbonyl groups serve as crucial biomarkers of oxidative stress [87]. The carbonyl content in proteins was quantified through spectrophotometric measurement, relying on the reaction between carbonyl groups and 2,4-dinitrophenylhydrazine, leading to the formation of 2,4-dinitrophenylhydrazone. The Protein Carbonyl Content Assay Kit (ab126287; Abcam, Cambridge, UK) was employed in accordance with the manufacturer’s instructions for assessing the carbonyl content. The following formula was utilized to express protein carbonyl content:

C = [(OD 375 nm)/6.364) × (100)] nmol/well

Protein Carbonyl Content = nmol carbonyl per mg protein = (C/P) × 1000 × D

where:

6.364 is the extinction coefficient using the enclosed 96-well plate in mM

C is the Carbonyl in your sample well (nmol)

P is the protein from the standard curve × 20 = μg/well

D is the dilution or concentration step applied to the sample

1000 is the factor to convert μg to mg

### 4.5. Assessment of Malondialdehyde and Lipid Peroxidation

Malondialdehyde (MDA) is a natural byproduct of lipid peroxidation (LPO). The measurement of MDA/LPO is essential for assessing oxidative damage to lipids. The determination of MDA concentration is based on its reaction as a byproduct of lipid peroxidation [88]. MDA undergoes a reaction with a reagent, such as thiobarbituric acid, resulting in the formation of a colored compound, particularly under acidic or high-temperature conditions [89]. The intensity of this colored product is measurable, and the optical density, or fluorescence, is directly proportional to the concentration of MDA. It is imperative to calibrate with standard solutions to ensure the accurate quantification of MDA levels in the sample.

The extent of LPO was assessed using a thiobarbituric acid reactive substance (TBARS) assay and measured via spectrophotometry. To do this, the TBARS Assay Kit (Item No. 10009055; Cayman Chemical, Ann Arbor, MI, USA) was employed following the manufacturer’s instructions.

The following method was utilized for expressing LPO: Calculate the average absorbance for each standard and sample. Subtract the absorbance value of Standard A (0 µM) from both the standards and samples to obtain the corrected absorbance. Plot the corrected absorbance values for each standard as a function of malondialdehyde concentration using Microsoft Excel 2019 (Version 16.0) Calculate the malondialdehyde value for each sample from the standard curve equation. The equation for calculating malondialdehyde concentration (μM) is as follows:

Malondialdehyde (μM) = [(corrected absorbance) − (y-intercept)]/slope

### 4.6. Detection of Antioxidative Enzyme Expression

The glutathione peroxidase (GPx) family of enzymes plays a crucial role in safeguarding organisms against oxidative damage. GPx is commonly assayed based on its ability to reduce a substrate, typically cumene hydroperoxide, while oxidizing reduced glutathione (GSH) to oxidized glutathione (GSSG) [90]. The generated GSSG is then reduced back to GSH with the consumption of NADPH, catalyzed by glutathione reductase (GR) [91]. The decrease in NADPH levels, measurable at 340 nm, is proportional to GPx activity. This principle allows for the quantification of GPx activity in various biological samples, providing insights into the cellular antioxidant defense system. GPx activity was assessed colorimetrically using the GPx Assay Kit (ab102530; Abcam, Cambridge, UK) as per the manufacturer’s instructions.

### 4.7. Protein Extraction and Western Blotting

Proteins were extracted from cultured ARPE-19 cells using radioimmunoprecipitation assay (RIPA) lysis buffer (20-188, Sigma-Aldrich, St. Louis, MO, USA) supplemented with 1% protease inhibitor cocktail and 1% phosphatase inhibitor cocktails 2 and 3 (Sigma-Aldrich). The protein concentration was determined using the bicinchoninic acid protein assay (Pierce, Rockford, IL, USA).

Electrophoresis was carried out using a 10% sodium dodecyl sulfate-polyacrylamide gel with 40 μg of the isolated protein. The obtained protein bands were subsequently transferred onto a polyvinylidene difluoride membrane (Immobilon-P; Millipore, MA, USA).

For immunoblotting, membranes were probed with the following primary antibodies: anti-nicotinamide adenine dinucleotide phosphate oxidase 2 (NOX2; 1:1000, ab80508, Abcam, Waltham, MA, USA), anti-immunoglobulin binding protein/glucose-regulated protein 78 (BiP/Grp78; 1:1000, 3177S; Cell Signaling Technology, Danvers, MA, USA), anti-inositol-requiring protein-1 (IRE1; 1:1000; ab37073; Abcam, Boston, MA, USA), anti-tumor necrosis factor-associated factor 2 (TRAF2; 1:1000; 4724S; Cell Signaling Technology, Danvers, MA, USA), anti-apoptosis signal-regulating kinase 1 (ASK1; 1:1000; ab131506; Abcam, Cambridge, UK), anti-B-cell lymphoma protein-2-associated X protein (Bax; 1:1000; 2772S; Cell Signaling Technology, Danvers, MA, USA), anti-B-cell lymphoma protein-2 (Bcl-2; 1:500; 26593-1-AP; ProteinTech Group, Rosemont, IL, USA), anti-caspase-9 (1:1000; ab25758; Abcam, Cambridge, MA, USA), anti-microtubule-associated protein 1A/1B-light chain 3B (LC3B; 1:1000; 2775S; Cell Signaling Technology, Danvers, MA, USA), anti-sequestosome-1 (P62; 1:1000; 5114S; Cell Signaling Technology, Danvers, MA, USA), anti-Beclin 1 (1:1000; SC-48341; Santa Cruz Biotechnology, Dallas, TX, USA), anti-phospho-adenosine monophosphate-activated protein kinase alpha subunit (phospho-AMPKα; Thr172; 1:1000; #2535; Cell Signaling Technology, Danvers, MA, USA), anti-adenosine mono phosphate-activated protein kinase (AMPK; 1:1000; #2532; Cell Signaling Technology, Danvers, MA, USA), anti-phospho-mammalian target of rapamycin (phospho-mTOR; Ser2448; 1:1000; #2971; Cell Signaling Technology, Danvers, MA, USA), anti-mammalian target of rapamycin (mTOR; 1:1000; #2983; Cell Signaling Technology, Danvers, MA, USA), anti-β-actin (1:2000; ab8226; Abcam, Cambridge, UK), anti-Vinculin (1:2000; ab129002; Abcam, Cambridge, UK), and anti-glyceraldehyde-3-phosphate dehydrogenase (GAPDH; 1:2000; MAB374; Millipore, Billerica, MA, USA).

Secondary antibodies used were anti-mouse immunoglobulin (Ig)G, horse radish peroxidase (HRP)-linked antibodies (#7076; Cell Signaling, Danvers, MA, USA), and anti-rabbit IgG HRP-linked antibodies (#7074; Cell Signaling, Danvers, MA, USA).

An enhanced chemiluminescence detection system (Pierce Biotechnology, Waltham, MA, USA) was used following the manufacturer’s instructions. Protein levels were quantified through densitometry analysis of the protein bands. Optical density levels were calculated and standardized based on the density of β-actin or a GAPDH band using ImageJ (version 1.54d; NIH, Bethesda, MD, USA).

### 4.8. Immunofluorescence Assays

Immunofluorescence assays were performed on ARPE-19 cells following the manufacturer’s instructions. In brief, the cultured ARPE-19 cells were fixed with 4% paraformaldehyde for 10 min. Subsequently, they were simultaneously blocked and permeabilized in a solution of 0.2% Triton X-100 and phosphate-buffered saline, containing 5% goat serum, for 20 min.

Following this, the cells were incubated at 25 °C for 1 h, followed by an overnight incubation at 4 °C with the appropriate primary antibodies diluted in the blocking solution. The primary antibodies used included anti-IRE1 (1:200) (ab37073; Abcam, Boston, MA, USA), anti-ASK1 (1:200) (ab131506; Abcam, Cambridge, UK), anti-cleaved caspase-3 (1:200) (#9661; Cell Signaling Technology, Danvers, MA, USA), anti-Beclin 1 (1:20) (SC-48341; Santa Cruz Biotechnology, Dallas, TX, USA), and anti-phospho-AMPKα (1:200) (#2535; Cell Signaling Technology, Danvers, MA, USA).

Subsequent to primary antibody incubation, appropriate fluorescent-labeled secondary antibodies were incubated in the blocking solution at 25 °C for 3 h. The cell nuclei were stained with 4′,6-diamidino-2-phenylindole (DAPI) for counterstaining.

Fluorescence intensity was measured using a microplate reader from Bio-Rad Laboratories. Protein expression levels were quantified using densitometry, and the relative density of immunostaining, represented as a fold change, was determined using an immunostaining index. The following formula was utilized for the densitometric quantitation of immunofluorescence, as previously outlined [92] with modifications:

Immunostaining index = Σ [(X-threshold) × area (pixels)]/total cell number

Here, X represents the staining density indicated by a grayscale value between 0 and 256, where X is greater than the specified threshold. In short, digital color images were acquired as PICT files and subsequently accessed in grayscale mode using the National Institutes of Health ImageJ Software (version 1.54d; NIH, Bethesda, MD, USA). Five images per experiment were analyzed and an average calculated. The relative density of immunostaining (fold change) was analyzed by an immunostaining index. As a reference, ARPE-19 cells cultured in NG medium were employed as control cells.

### 4.9. Detection of Apoptosis through Terminal Deoxynucleotidyl Transferase dUTP Nick end Labeling Assay

The identification of apoptosis through the Terminal Deoxynucleotidyl Transferase dUTP Nick End Labeling (TUNEL) assay is a widely employed technique in molecular and cellular biology [93,94]. This technique allows for the visualization and quantification of apoptotic cells by identifying DNA fragmentation, which is a hallmark of apoptosis.

A TUNEL assay was conducted using the FragEL^TM^ DNA Fragmentation Detection Kit, Colorimetric (QIA-21, Calbiochem, Darmstadt, Germany) following the manufacturer’s provided guidelines. In the TUNEL assay, TdT is used to add labeled dUTP (deoxyuridine triphosphate) to the 3’-OH ends of fragmented DNA in apoptotic cells. In this assay, fluorescent signals were captured subsequent to the introduction of fluorescein isothiocyanate (FITC)-avidin, which specifically binds to the biotinylated-deoxyuridine residues present in damaged DNA.

The staining images obtained from this process were subjected to analysis to determine and quantify the relative fluorescence intensity of TUNEL-positive cells. This assay is a valuable method for identifying and assessing cells undergoing DNA fragmentation, a characteristic feature of apoptosis.

### 4.10. Assessment of Mitochondrial Dysfunction by Assessing Changes in Mitochondrial Membrane Potential

Mitochondrial membrane potential was assessed using the JC-1 Mitochondrial Membrane Potential Assay Kit (10009172, Cayman Chemical Company, Ann Arbor, MI, USA). The JC-1 Mitochondrial Membrane Potential Assay offers a powerful means to assess mitochondrial function by distinguishing between healthy and unhealthy cells based on changes in membrane potential [95]. This procedure involved the following steps:

ARPE-19 cells were seeded into 96-well plates at a density of 1 × 10^4^ cells per well. After seeding, the cells were allowed to incubate at 37 °C for 24 h, providing sufficient time for them to adhere and stabilize. Subsequently, add 50 µL of JC-1 staining buffer to 1 mL of the culture medium. JC-1 is a unique dye that forms J-aggregates in healthy cells with functional mitochondria, while it remains in its monomeric form in apoptotic or unhealthy cells. To quantify the changes in mitochondrial membrane potential, the fluorescence intensities of J-aggregates (stained with Texas Red, indicating healthy cells) and JC-1 monomers (stained with FITC, indicating cells undergoing apoptosis or exhibiting unhealthy characteristics) were measured using a microplate reader provided by Bio-Rad Laboratories. These two fluorescence channels have distinct excitation/emission wavelengths: 560/595 nm for Texas Red and 485/535 nm for FITC. For the final step, the number of cells displaying either red or green staining (representing healthy or apoptotic/unhealthy cells, respectively) was counted in a blinded manner. This cell counting and fluorescence analysis were carried out using ImageJ (version 1.54d; NIH, Bethesda, MD, USA).

### 4.11. Statistical Analyses

The data were expressed as mean ± standard deviation values. To assess differences between groups, Student’s t-tests were employed, and for multiple comparisons, a one-way analysis of variance (ANOVA) or Kruskal–Wallis test followed by a post hoc analysis (Bonferroni test) was performed. Statistical significance was considered at *p* < 0.05. All statistical analyses were carried out using SPSS for Windows (version 19.0; IBM, Armonk, NY, USA).

## 5. Conclusions

**Figure 7:**
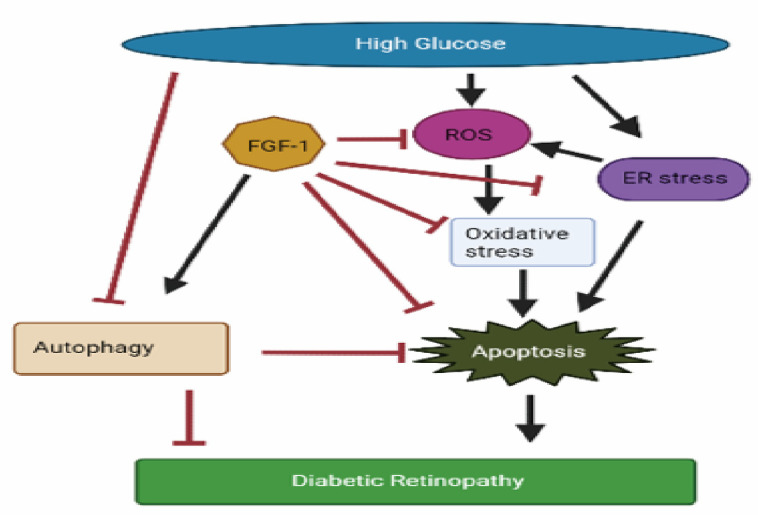
FGF-1 protects retinal pigment epithelial cells from high glucose-induced damage: alleviation of oxidative stress and endoplasmic reticulum stress, and restoration of autophagy.

Our study reveals that HG induces elevated oxidative stress and ER stress, culminating in apoptosis in ARPE-19 cells. Early intervention to mitigate HG-induced ER stress and apoptosis and to enhance cell survival emerges as a critical strategy for safeguarding retinal cells against DM-related damage. FGF-1 demonstrates a significant capacity to alleviate HG-induced oxidative stress and ER stress, subsequently reducing apoptosis through the enhancement of AMPK/mTOR signaling to restore autophagy (Figure 7). These findings underscore the effectiveness of FGF-1 as a promising candidate for the treatment of diabetic retinopathy, providing a potential avenue for therapeutic intervention.

## Data Availability

The data that support the findings of this study are available on reasonable request from the corresponding author.

## Supplementary Materials

The following supporting information can be downloaded at: https://www.mdpi.com/article/10.3390/ijms25063192/s1.
