# Supplementary figures and images for: Beneficial Effects of Fibroblast Growth Factor-1 on Retinal Pigment Epithelial Cells Exposed to High Glucose-Induced Damage: Alleviation of Oxidative Stress, Endoplasmic Reticulum Stress, and Enhancement of Autophagy
Source: Int J Mol Sci. 2024 Mar 11;25(6):3192. doi: 10.3390/ijms25063192 (PMC10970413; doi:10.3390/ijms25063192)

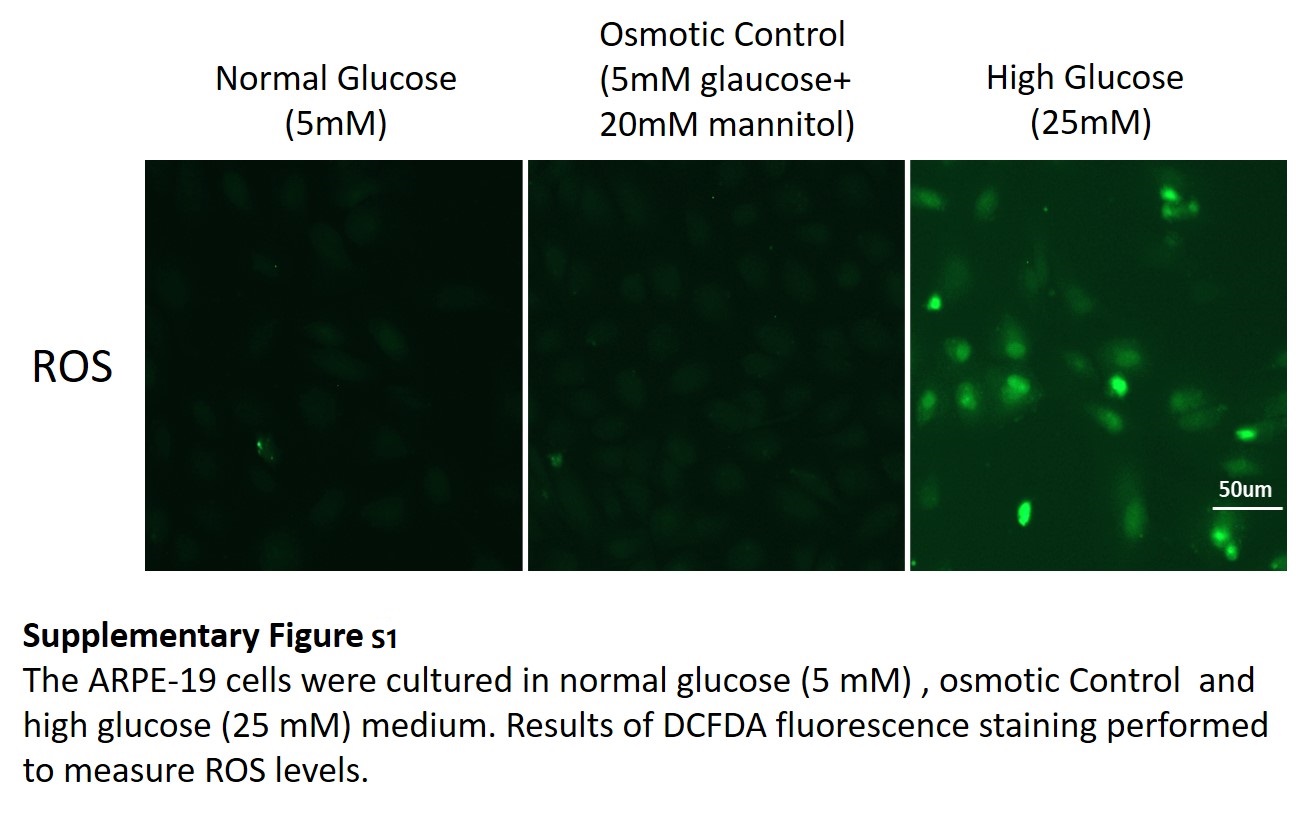

Supplement: Supplementary file 1 [file ijms-25-03192-s001.zip › IJMS Supplementary Figure S1.jpg]

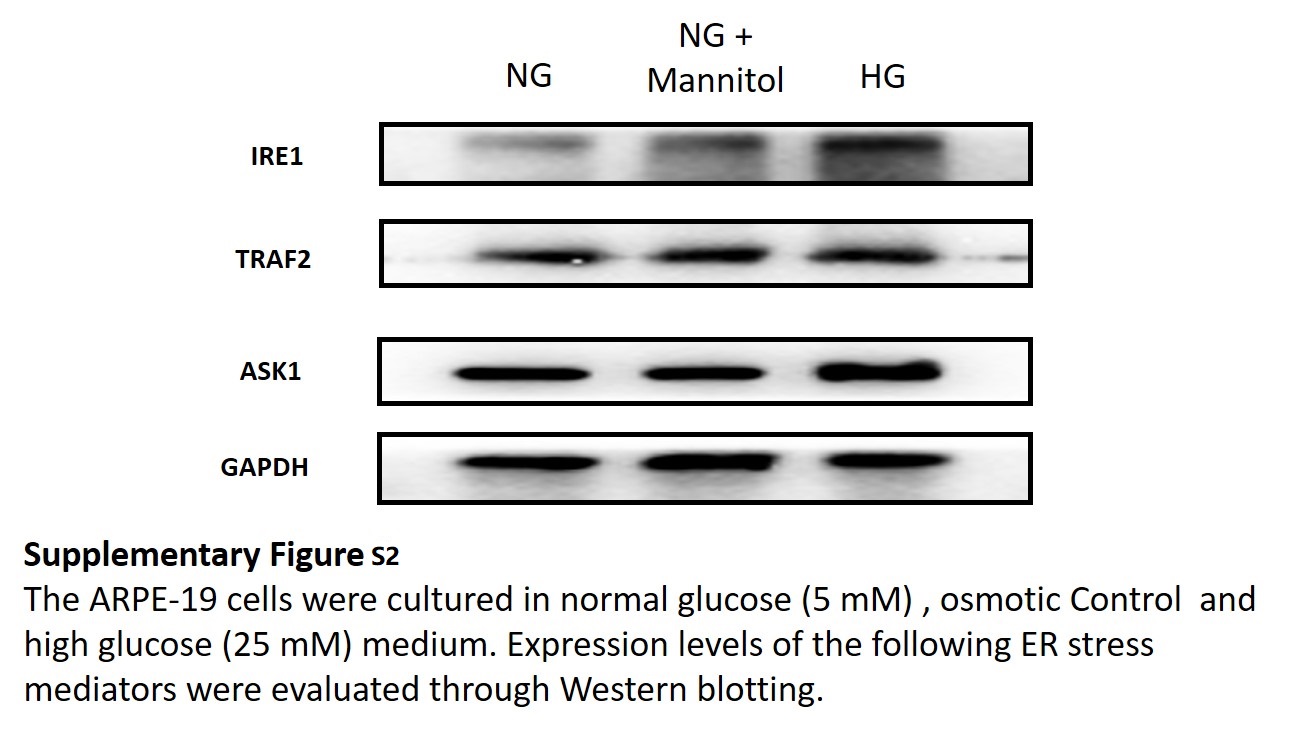

Supplement: Supplementary file 1 [file ijms-25-03192-s001.zip › IJMS Supplementary Figure S2.jpg]

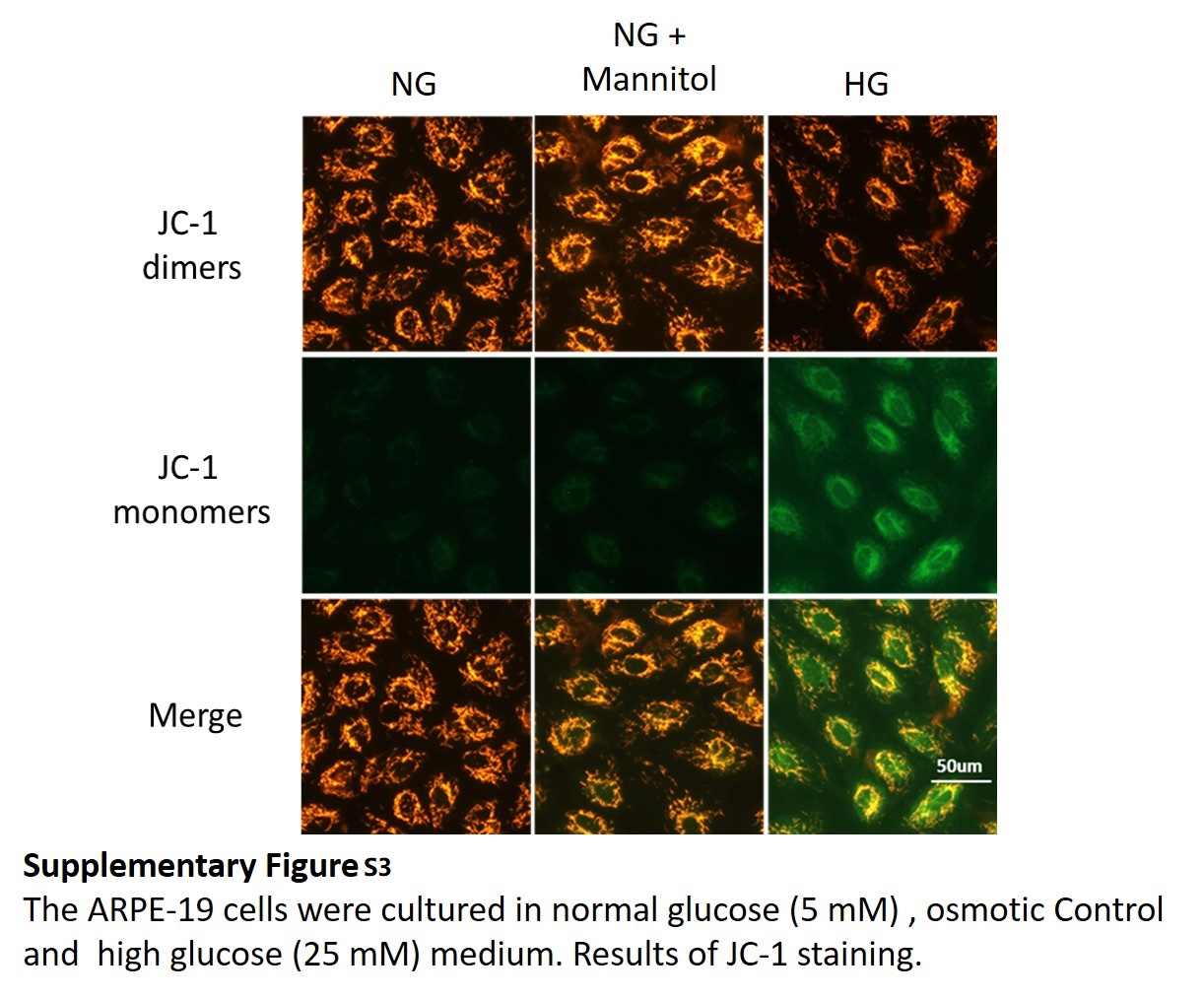

Supplement: Supplementary file 1 [file ijms-25-03192-s001.zip › IJMS Supplementary Figure S3.jpg]
